# Intrasplenic Pancreatic Pseudocyst after Chemoradiation of a Pancreatic Adenocarcinoma Mimicking Progressive Disease: A Case Report and Review of the Literature

**DOI:** 10.1155/2019/5808714

**Published:** 2019-02-14

**Authors:** Thomas Benter, Oliver Roehr, Lutz Moser, Philipp Kiewe, Leopold Hentschel, Ivan Platzek, Markus K. Schuler

**Affiliations:** ^1^Klinik für Onkologie, Helios Hospital Emil von Behring, Berlin, Germany; ^2^Praxis für Implantologie und ästhetische Zahnmedizin, Traunstein, Germany; ^3^MVZ Strahlentherapie am Helios Klinikum Emil von Behring, Berlin, Germany; ^4^Onkologischer Schwerpunkt am Oskar-Helene-Heim, Berlin, Germany; ^5^Universitäts KrebsCentrum, Universitätsklinikum Carl Gustav Carus, Dresden, Germany; ^6^Klinik für Radiologie, Universitätsklinikum Carl Gustav Carus, Dresden, Germany

## Abstract

Chemoradiation is one of the therapeutic options in palliative treatment of locally advanced pancreatic adenocarcinoma, with a well-known safety profile. In this case report, we describe the treatment-related occurrence of an intrasplenic pancreatic pseudocyst which was successfully removed by gastrocystic drainage. This rare complication should be considered in the follow-up and clinical management of patients, particularly if left-sided complaints occur.

## 1. Introduction

There is broad evidence for chemoradiation either as primary treatment or consolidation of locally advanced or unresectable pancreatic cancer [1, 2]. Chemoradiation primarily achieves to delay local progression and to prevent frequently accompanying symptoms such as pain. An effect on overall survival, however, has not been proven [3]. Compared to gemcitabine monotherapy, simultaneous chemoradiation may cause more grade 4 adverse events, mainly anemia, neutropenia, thrombocytopenia, fatigue, nausea, and vomiting [4]. However, specific, severe, and potentially fatal local adverse events caused by chemoradiation of pancreatic tumors have rarely been reported. These include gastrointestinal ulcerations or duodenal stenosis within the high-dose area of radiation with an estimated dose-dependent risk between 2 and 5% [5]. Pancreatic pseudocysts are known to be caused by chronic pancreatitis, trauma, and surgery [6]. Splenic involvement has been reported as a rare complication in acute and chronic pancreatitis with an incidence of 2% in acute pancreatitis [7–9]. Pancreatic pseudocysts may also develop as a result from occlusion of the main bile duct caused by pancreatic adenocarcinoma [10]. However, the development of a pancreatic pseudocyst as complication of systemic or local treatment of pancreatic cancer has not yet been reported. For the management of pancreatic pseudocysts, endoscopic drainage has among others become a valuable alternative to surgical procedures [11].

## 2. Case Report

A 74-year-old woman who suffered from pancreatic adenocarcinoma of the corpus, including peritoneal and bone metastases, had received palliative chemotherapy with gemcitabine for eight months; however, she switched to concomitant chemoradiation due to painful symptomatic primary tumor progression. Treatment consisted of percutaneous modulated arc radiotherapy with single doses of 3.0 Gy five times a week up to a total dose of 36.0 Gy. The planning target volume was 102 ccm. Radiation was combined with fluorouracil (225 mg/m^2^/d) as continuous infusion.

**Figure 1:**
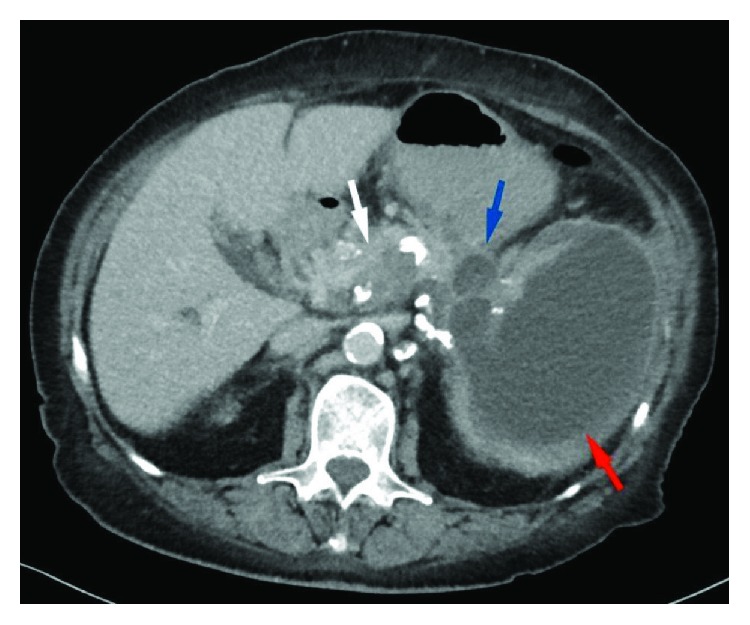
Axial contrast-enhanced computed tomography (CT) image. A large hypointense pseudocyst fills out nearly the entire spleen (red arrow), while a smaller component of the pseudocyst is located in the pancreatic tail (blue arrow). The already histologically proven adenocarcinoma is partially visible as a thickened and hypodense area of the pancreatic corpus (white arrow).

Four weeks after the completion of chemoradiation, the patient presented in the emergency room with vomiting and rapidly increasing pain in the upper left abdomen, and gastrointestinal obstruction due to progressive disease was suspected. Computed tomography (CT) and magnetic resonance imaging (MRI) scans showed an intrasplenic cyst (Figure 1) with a size of 14 × 13 × 16 cm. Fine needle aspiration revealed mesothelial cells and elevated levels of lipase but no tumor cells. Therefore, the diagnosis of an intrasplenic pancreatic pseudocyst was made. There were no signs of a splenic rupture or peritonitis. Because of the massive painful enlargement and the risk of intraperitoneal rupture, we performed a gastrocystic drainage from the cardia into the upper part of the intrasplenic cyst. The technique was undertaken with a short needle path, with less splenic tissue between the gastric wall and the cyst, using endosonography to place a 4 cm double pigtail. The pigtail drainage produced brown cloudy liquid without the presence of any tumor cells.

**Figure 2:**
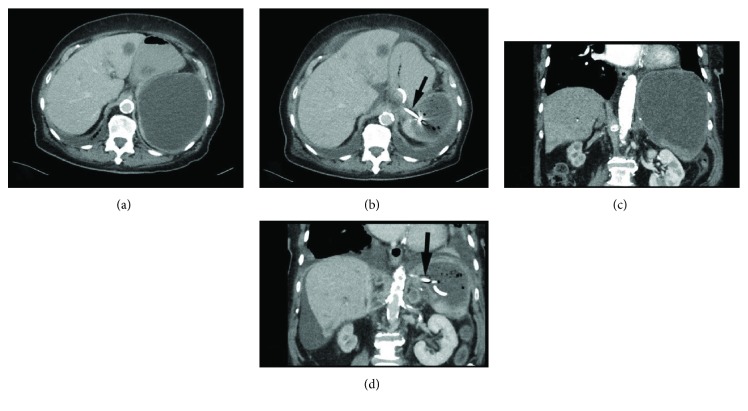
Axial (a) and coronal (c) contrast-enhanced CT images of the splenic pseudocyst before drainage placement and corresponding CT images (b, d) two days after the placement of the gastrocystic drainage. The pigtail drainage is marked with a black arrow. Note the reduction of size of the pseudocyst after drainage placement. A hypodense liver metastasis is seen in the left liver lobe in both axial images.

Within the following days, the patient experienced relief from pain and had bowel movements. A CT scan and ultrasound showed shrinkage of the cyst and air in the parenchyma of the spleen as the organ returned to its typical shape (Figure 2). Free intra-abdominal air was not detected. The patient recovered without further pain in the upper left abdomen; however, she died six weeks later because of the progressive systemic disease.

## 3. Discussion

Pancreatic cancer may unexpectedly occur with pancreatic pseudocysts related to chronic pancreatitis [12]. Also, intrahepatic pancreatic pseudocysts have been reported as uncommon manifestations of pseudocysts [13]. Moreover, pancreatic cancer can be accompanied or masqueraded by a pseudocyst [14]. However, in the case reported herein, this was very unlikely because CT imaging was performed prior to chemoradiation and several times during systemic treatment and did not reveal any cystic lesions. Neither did the patient express any complaints related to tumor mass before. Thus, to the best of our knowledge, this is the first report describing a pancreatic pseudocyst as a complication of chemoradiation to a pancreatic adenocarcinoma.

In a series of 129 patients presenting with pancreatic pseudocysts at a large tertiary cancer center over a period of ten years, no report of a cancer- nor treatment-related pseudocyst exists [15]. Moreover, a thorough literature search did not reveal any case of chemoradiation-induced pancreatic pseudocyst. Nevertheless, we cannot completely exclude other causes like progressive disease. Pancreatic ductal adenocarcinomas with cystic features as a diagnostic pitfall have been reported [16], but it seems to occur rather rare [17]. They can also arise from intraluminal obstruction of the pancreatic duct, which again we did not observe in our patient.

We were able to successfully alleviate symptoms caused by the pseudocyst using an endosonographic internal drainage system that is nowadays a valuable option for the treatment of symptomatic pancreatic pseudocysts [15]. This technique has been widely adopted in the management of pancreatic pseudocysts [18, 19].

Ultimately, adequate imaging and careful clinical investigation are recommended in patients with chemoradiation during treatment and follow-up in order to prevent discontinuation of therapy due to pseudoprogression. Furthermore, the risk of developing a symptomatic pseudocyst should be discussed by the treating physician prior to chemoradiation of a pancreatic tumor.
